# The analysis of dynamic emotional contagion in online brand community

**DOI:** 10.3389/fpsyg.2022.946666

**Published:** 2022-09-15

**Authors:** Dewen Liu, Sikang Zhang, Qi Li

**Affiliations:** ^1^School of Management, Nanjing University of Posts and Telecommunications, Nanjing, China; ^2^College of Business, Shanghai University of Finance and Economics, Shanghai, China; ^3^School of Economics and Management, Tongji University, Shanghai, China

**Keywords:** online brand community, emotional contagion, posts, feedback, sentiment analysis

## Abstract

Online brand communities (OBCs) could benefit firms in many usages, ranging from collecting consumers’ suggestions or advice to interacting with community members directly and transparently. Creating a positive emotional atmosphere is essential for such communities’ healthy development as its boosts the continuous involvement of each member. However, the dynamic cross-influences and evolution of emotions in OBCs have not been fully explored, which was the research gap this paper tried to fill. Based on emotional contagion theory, this study identifies three sources of textual sentiment through machine learning methods in OBCs: member’s posts, other members’ feedback, and the focal firm’s official feedback. This study further tested the dynamic emotional contagion process among these sources on valence (mean) and volatility (dispersion), namely how they affected each other. Data was collected from the MIUI forum, a large forum launched by Xiaomi corporate on August 1, 2011, which contained 17,622 posts and 99,426 feedback. Results showed that: (1) in the emotional contagion process, there existed differences in the influence of emotional valence and volatility from different sources; (2) all emotional interactions were temporary and mostly lasted no more than three days; (3) the most significant contributor of each sources’ emotion was itself, which could be explained by lagged effect; (4) the valence of focal firm’s emotion (focal firm’s official feedback) was the second contributor of the valence of member’s emotion (member’s posts) and other members’ emotion (other members’ feedback). Three sources of emotion in OBCs and emotional valence/volatility should be considered when firms try to guide the emotional changes in such communities. Furthermore, firms could proactively influence members’ emotions by carefully designing the feedback to members’ posts. Besides, since all interactions are temporary, firms need to engage in online communities frequently, like consistently offering feedback.

## Introduction

An online brand community (OBC), such as Apple Support Communities and Dell IdeaStorm, is a group of consumers with a shared enthusiasm for the brand and a well-developed social identity, whose members engage jointly in group actions to accomplish collective goals or express mutual sentiments and commitments (Bagozzi and Dholakia, 2006; Demiray and Burnaz, 2019). OBCs have a wide range of essential usages which benefit both consumers and the firms that launched them. For example, the placeless and timeless nature of OBCs provides easy access to product-related information exchange. It facilitates long-term relationship building and maintenance between consumers and firms, which helps them get optimal product solutions quickly through posting online (Palmer and Koenig-Lewis, 2009). For firms, OBCs provide new conduits or channels to advertise and promote their new products (Wu et al., 2018), communicate with or educate consumers (Goh et al., 2013; Cao et al., 2021), boost consumers’ involvement or disseminate positive Word of Mouth (Hsieh et al., 2022), and increase the customer base for the firm (Schau et al., 2009; Popp and Woratschek, 2017). Accordingly, firms can proactively take advantage of OBCs to detect, prevent, and respond to adverse events (Herhausen et al., 2019), increase consumers’ loyalty by improving experiences through support programs (Huang et al., 2015), or even directly collect community members’ posts to promote open innovations and new product adoption (Bayus, 2013; Huang et al., 2014; Yang and Wang, 2017). As an example, Xiaomi, a globally leading global manufacturer of smartphones, has many functional improvements in its new products stem from members’ posts in their OBC: MIUI. Xiaomi has also used this community to create a loyal group of consumers who continue to spontaneously purchase and buzz their products.

To correctly and effectively manage OBCs and creat a harmonious atmosphere, firms cannot ignore one of the critical factors in OBCs: emotions (Daradkeh, 2021; Villa et al., 2021; Liu, 2022). Unlike other non-brand-related online communities (e.g., microBlog communities), OBCs are brand-leaded and gather consumers with similar experiences (Zheng et al., 2022). Therefore, this kind of community organized by loose relationships relies more on the harmonious sentimental atmosphere to promote the members’ continuous involvement (Nadeem et al., 2020). They can easily switch to other communities or even drop out if they sense negative or unstable emotions in OBCs (Liao et al., 2020). Many studies have confirmed that emotions can influence or predict members’ behaviors in OBCs and highlighted the role of emotion in community management practice. For example, Gu et al. (2014) found that the Yahoo Finance community members are more likely to reply to posts with similar emotions. Yan and Tan (2014) confirmed that the emotional support that patients received in online health communities helps them to improve their health status. In addition, studies confirmed that members’ emotions toward a brand or product in third-party communities (e.g., Twitter) could be applied to predict customer satisfaction (Situmeang et al., 2019) and overall sales (Lau et al., 2018). Thus, the importance of emotions in the online environment has been advocated by current research. However, in contrast to traditional virtual communities where only members participate, OBCs’ related parties include members and the firm’s employees. As representatives of the firm, the participation of these staff is their daily work, and their replies to members’ posts or their interactions with members represent the firm’s official intentions. Therefore, the emotional contagion process in OBCs must be different from that in other online communities since it includes the involvement of official staff. Besides, most studies treated emotion as an essential antecedent to predict other marketing outcomes (e.g., satisfaction, sales), but have neglected empirically the vital research proposition of influencing and managing community emotions. Although scholars (e.g., Casaló et al., 2009; Cheng et al., 2020; Yuan et al., 2020) have recently paid attention to the particularity of emotion in OBCs, current literature remains two unresolved questions that have not been fully answered yet: (1) What are the sources of mutual influences of emotions in such community? (2) What can the firm do to influence the members’ emotions to achieve a harmonious atmosphere. This study approaches these questions empirically with an explanation based on the emotional contagion theory.

Emotional contagion theory provides us a applicative lens to investigate the mutual emotional influences in OBCs. Existing literature on emotional contagion theory has demonstrated that people tend to mimic others in emotional aspects during the interpersonal interaction process (Hatfield et al., 1993); that is, if people quarrel with others, they will also feel angry even though they do not have such negative emotions at the beginning. This phenomenon also holds in virtual contexts (e.g., social media: Coviello et al., 2014). Therefore, according to the emotional contagion theory, an emotional contagion process exists in OBCs, leading firms to influence members’ emotions proactively. However, the existing literature has failed to investigate this potential dynamic emotional contagion process, especially lacking a holistic understanding of the role of the firm’s emotion, reflected by the staff’s feedback on members’ posts, which hinders the firm’s practical managerial actions and fails to provide operable guidance on how to “orchestrate” their own emotion to influence members’ emotions in OBCs. In sum, this study takes the posts with replies on the MIUI forum from 2013 to 2014 as research units and applies the emotional contagion theory to explain the emotional dynamic influence process. Based on the VAR model, impulse response function analysis, and VAR model’s variance decomposition, the sources of emotion in this OBC were divided into three: members’ posts, other members’ feedback, and the focal firm’s official feedback (the staffs’ feedback). Then, the study examined the directions of mutual influences and contribution rates of the three sources on the emotional mean (reflects valence) and dispersion (reflects volatility) in the subsequent ten periods.

The possible contributions of this study are as follows. First, to the best of our knowledge, this paper is the first to distinguish the different sources of emotion in OBCs, considering the firm’s and other members’ roles simultaneously, thus contributing to the current OBCs research. Second, this study hypothesized that emotional valence and volatility could be contacted in the emotional contagion process simultaneously, and emotions from different sources have different effects on each other in this process, expanding the trial scope of emotional contagion theory. Third, in practice, this paper aimed to study how the valence and volatility of the firm’s emotions affect other different sources’ emotions (member’s posts and other members’ feedback in OBCs), and analyzed the duration of the impact and the corresponding rates of contribution, which provides helpful guidance on how firms can actively influence the emotions of community members in OBCs to manage the community effectively.

The rest of this paper is organized as follows: First, we reviewed the emotional contagion literature and its application in the online environment, introducing the current status and progress of the stream of research. Next, this paper will describe the data and the process of measuring the emotional valence and volatility of three sources. In the following section, empirical results will be shown. Finally, contributions, implications, and limitations will be discussed in the end.

## Literature review

Emotions are feelings generated from both conscious and unconscious processing. A specific emotional assessment of a situation is a general evaluation of that situation (whether positive or negative), manifesting in mental and bodily responses (Wright, 2002). In early work about emotions, Hatfield et al. (1993) firstly summarized the commonalities of human-to-human chats and talks. They found that people would automatically imitate and synchronize other people’s facial expressions, voices, and postures and then make emotional convergence happen. They defined this phenomenon as emotional contagion. Emotions can be transmitted from one individual to another. For example, Hsiao et al. (2022) found that restaurant servers and customer influence each other’s emotions through verbal and behavioral interactions. Yu et al. (2022) further indicated that the bystanders’ emotional status are also influenced by what they observe and by the interpersonal interactions of others.

To firms, consumers’ emotions are indirect motivators of purchase behavior, shape brand saliency, and influence attitudes, beliefs, inclinations, and perceptions (Morrison and Crane, 2007). Akdim (2021) argued that sentimental analysis could help the firm improve the quality of products/services, identify customer needs, and implement new marketing strategies. The effectiveness of this analysis also holds across various cultures and countries (Kusawat and Teerakapibal, 2022). In OBCs, the emotions beneath online conversations decide the consumers’ reactions to active firms’ actions (Homburg et al., 2015). The neural analyses (through electroencephalography) showed the valence of the arousal, which indicates that emotional valence of online consumer review activate arousal and emotional status in the observer (Herrando et al., 2022). However, little work has been done in conducting sentimental analysis in OBCs setting (e.g., Situmeang et al., 2019), especially the cross-influences from different sources. In OBCs, when a member puts a post, other members and the firm’s staff would make replies. In their interaction, each party’s emotions may be affected by other parties.

Existing literature also conducted in-depth explorations of the reasons behind the occurrence of emotional contagion. Wang et al. (2010) showed that the possible mechanism of emotional contagion is imitation→feedback; that is, emotional subjects imitate other people’s intonation and actions and then get feedback from imitation to express emotion. Zhang and Lu (2013) pointed out in more detail that the mechanism of emotional contagion is: perception→[imitation→feedbacks (activate the mirror nervous system)] → emotion, that is, the subject perceives and imitates the emotion of other people and gets feedback from the imitation, and finally shows the personal emotion. Emotional contagion is a bi-directional influence process (Liu et al., 2022) and each party influences the other in a dynamic relationship of influence (Luo et al., 2022). Emotional contagion is a ubiquitous phenomenon that occurs in many scenarios and is not limited to face-to-face or conscious interactions (Tajmirriyahi and Ickes, 2022; Xie et al., 2022). For example, in an experimental study, the subjects were asked to watch pictures of happy faces, and these subjects showed pleasant emotions later (Lundqvist and Dimberg, 1995). The positive emotion of individual animals would be transmitted to the group (Reimert et al., 2013). The investors’ emotions in one country can even spread to investors in another country (Yin and Wang, 2020). Thus, this research argues that this mutual influence process of emotions would also occur in OBCs, which is manifested in the textual posts and replies.

Although there are relatively few studies about emotions in OBCs, the existing literature has fully confirmed that emotional contagion can also occur in the online environment. For example, Kramer et al. (2014) conducted an experiment based on Facebook and found that if content with fewer positive words is pushed to users, users will reduce the use of positive words and use more negative words when publishing new content. Lin and Utz (2015) also studied the phenomenon and boundary conditions of emotional contagion on Facebook. They found that when the contents that the user browses are positive and come from a closer relationship (e.g., family members), they will express more positive emotion in subsequent content creation. Liu et al. (2022) confirmed that the each parties’ linguistic styles can be influenced by others in Xiaomi community. In a similar context, Crocamo et al. (2021) gathered 3,308,476 tweets and found negative sentiment proportion of tweets gradually increased with amplifications following key events (e.g., the naming of COVID-19 on February 11, 2020). Of particular relevance to this study, Lee and van Dolen (2015) investigated the role of the firm’s emotion, reflected by the words the official staffs use, in the emotional contagion process and found the more positive words the official staff used toward members in online communities, the more positive their own emotion reported by members. Homburg et al. (2015) and Meire et al. (2019), based on ten online forums and Facebook, respectively, also found that the feedback released by official staff to users can affect users’ emotions, so it is logical to infer that this emotional contagion will also take place in OBCs because of the shared features (e.g., anonymity, virtualness) of OBCs and the above studies’ contexts. Flavián et al. (2011) found that online searches influence users’ emotional states because of the emotions experienced during the search process and the effort they have to find the information they are looking for or their perception of success. Similarly, when members post or reply, they also experience emotions as they participate in the community and show this in their textual expression.

In sum, existing literature on emotional contagion and OBCs management reveals the possibility of emotional emergence. However, existing literature has not focused on the cross-interactions of emotional valence and volatility from different sources. Specifically, this study tries to fill the following two gaps in existing research.

First, from the perspective of the sources of emotions, although some studies distinguish the two sources of emotions: members and firm, and explore the impact of firms’ emotions on members’ emotions (e.g., Lee and van Dolen, 2015), they have not addressed the sources of other members’ emotions. Posts’ feedback is as much a part of the post as it reflects the interactivity between posts and feedback, which stands for the conflict or resonance of views and emotions. Thus, there may be heterogeneity in the emotions between posts and responses as they naturally represent the two parties involved in the conversation. The spread of emotions in OBCs is an essential factor influencing the overall emotional status. For example, the spread of negative emotions between main posts and feedback or between firms’ feedback and other members’ feedback may lead to the loss of control of online public opinion in the context of information uncertainty and trigger a series of adverse social effects (Kramer et al., 2014; Crocamo et al., 2021). Accordingly, this paper argues that further distinction in detecting the detailed cross-influence is necessary. In OBCs’ practice, some firms may prefer members to post more to get ideas to improve products, and others may want members to post and reply to other posts proactively and simultaneously to form a communal community identity between community members. Although these firms have different purposes in managing OBCs, they are consistent in how to motivate their members to form relatively stable positive emotions to involve in posting and replying. Therefore, further distinguishment of the sources of community members’ emotions into posters and repliers can reveal the influence of the firm’s emotions on members’ emotions in a more detailed way and provide helpful guidance on how the firm’s emotions affect members’ emotions from different emotional “outlets.”

Second, the existing studies mainly detect the role of emotional valence, which failed to examine the role of emotional volatility in the emotional contagion process. From a holistic view, emotional valence and volatility will appear simultaneously, concealing the difference between textual data with the same valence. For example, when a community member puts a post about product pros (positive emotions) and another community member puts a post about product cons (negative emotions). Although the emotion valence could be relatively low due to the offset, the emotional content of the textual data is still rich, reflected in the emotional volatility. Consequently, it is more realistic to examine the role of emotional valence and volatility in emotional contagion synchronously. In addition, existing research failed to examine the duration and contribution rates of the mutual influence of emotions from different sources and cannot help the firm judge the duration and weight of influence of the impact of specific management actions in OBCs, which leaves a significant gap that this paper tries to fill.

## Materials and methods

### Data

The original data of this paper stems from the new function discussion section, which belongs to the MIUI forum (https://www.xiaomi.cn/), an OBC established by Xiaomi Corporation on August 1, 2011, for consumers to discuss the operating system and related products. Xiaomi is a leading designer and manufacturer of consumer electronics (e.g., mobile phones) and related software, home appliances globally. The MIUI forum was created for its “fans” to communicate with each other, interact with the firm and participate the official activities. As a result, choosing this forum for research has certain representativeness. Up to December 5, 2015, the MIUI forum has exceeded 30 million members, with an average daily posting volume of more than 100,000 and a cumulative posting total of more than 200 million. It has been used as the context for many consumer psychology studies (e.g., Xiang et al., 2022). In the MIUI forum, anyone can register and become a community member. They can post to discuss issues related to the MIUI system/Xiaomi products or make suggestions. For those members who purchase Xiaomi products, the corresponding product icons will be officially lit up on their homepage.

Through crawling the posts and feedback in this OBC, this paper collected the following data: posts data includes the identity of the posters, the time of posting, and the content of the posts; and feedbacks data of the posts, including the content of feedbacks, the identity of the replier, and the replied time of each feedback. Among them, according to the identity of repliers, this divided the feedback into other members’ feedback and the firm’s feedback to measure other members’ and firm’s emotions, respectively. In this study, the originator of a post is defined as the poster, and all replies below this post are considered repliers. It should be noted that posters sometimes participate in replies to posts, but these posts are not identified as replies in the study of this study.

The time range of the original data is from January 1, 2013, to December 31, 2014. During this period, Xiaomi has made two major upgrades to the MIUI system: MIUI V5.0 was released on April 9, 2013, and MIUI V6.0 was released on August 16, 2014. To avoid the possible confounding influence before and after the release of the new version system (e.g., the behavioral changes due to maladjustment to the new system), this paper finally selected data from two months after the release of MIUI V5.0 to two months before the release of MIUI V6.0, a total of 373 days, and the time range is from June 9, 2013, to June 16, 2014. Finally, because the emotional contagion process in this paper is dedicated to the influence of members’ emotions, only 58 posts were released by the firm, which is a small portion. Then we removed these 58 posts released by the firm and its feedback. In the final data, the number of posts was 17,622, the number of firm’s feedback was 9,431, and the number of other members’ feedback was 89,995.

### Measurements

A machine learning approach was used to calculate the textual emotion (both posts and feedback), which avoids the potential bias of judging emotion by artificial scoring in previous literature. The software this paper use is Python. First, this paper referred to the dictionary method introduced by Lin and Xie (2017) to calculate the emotion of the textual content. The calculation formula of the emotion of the text is as follows:


(1) Pos=Numbersofpositivewords/Totalnumbersofwords

(2) Neg=Numbersofnegativewords/Totalnumbersofwords

(3) $$\mathrm{Textual}\;\mathrm{emotion}=\frac{\left(Pos-Neg\right)}{\left(Pos+Neg\right)+0.0001}$$

It should be noted that this paper adds 0.0001 to the denominator of Equation (3) to deal with the case where the denominator is zero and prevent computational mistakes, which is suggested by Xiang et al. (2022).

Secondly, consistent with prior literature, the Jieba library in python was used to complete Chinese word segmentation (Hu and Wang, 2021). Again, this paper referred to prior literature and merged the following three words to build the final dictionary. These three dictionaries are the HowNet Sentiment Dictionary (Zhang and Zhou, 2020), National Taiwan University “Chinese Sentiment Polarity Dictionary” (Li et al., 2019), and “The Chinese Commendatory and Derogatory Dictionary” (Wu et al., 2009).

Finally, compare the results of text segmentation with the dictionary to get the number of positive and negative words in each text, and then combine with Equation (3) to calculate the textual emotion, which provides the foundation for the calculation of emotional valence and volatility in any given day.

This paper divided the sources of sentiment into three parts: member’s posts, other members’ feedback, and the focal firm’s feedback, and then calculated the valence and volatility of each source’s sentiment. This paper used the standard deviation of different textual emotional values to measure emotional volatility on a given day. Combined with the textual preparation process introduced above, this paper defined the following six variables: three sources’ emotional valence and volatility. For example, this paper used m_pt to denote the emotional valence (mean) of all posts on day t, which represented that we calculated the mean value of all posts based on Equation (3) on any given day. In the same vein, the detailed definitions and descriptive statistics of the variables are presented in Table 1. In the following part, this paper will construct a vector autoregressive (VAR) model for the mutual influence of these variables and report the forecast error variance decomposition (FEVD) results. The VAR model is a stochastic process model that has been widely used to capture the causal relationship between multiple quantities as they change over time. In our research context, the emotions from different sources affect each other in a dynamic manner as emotional contagion suggested. Compared to other methods (e.g., linear regression), the VAR approach can better capture the mutual dynamic effects highlighted in this theory (Bringmann et al., 2018). Thus, the VAR approach was adopted in this study.

**Table 1:** Definition and descriptive statistics.

Variable	Interpretation	Mean	SD.	Min	Max
$m\_pt_{t}$	Mean value of emotion of all posts on day t	0.252	0.092	−0.038	0.516
$m\_uct_{t}$	Mean value of emotion of all other members’ feedbacks on day t	0.244	0.059	0.066	0.413
$m\_\mathrm{fc}t_{t}$	Mean value of emotion of focal firm’s feedbacks on day t	0.203	0.211	−1	1
$v\_pt_{t}$	Standard deviation of emotion of all posts on day t	0.550	0.059	0.363	0.782
$v\_uct_{t}$	The standard deviation of the emotion of all other members’ feedback on day t	0.642	0.027	0.558	0.721
$v\_\mathrm{fc}t_{t}$	The standard deviation of the emotion of the focal firm’s feedback on day t	0.595	0.136	0	1.414

## Var model construction

### Stationarity test

Before conducting a VAR model, it is first necessary to check whether the variables are stationary. In this paper, the first-order difference processing was performed on each variable. The first-order difference function is the value of a variable in the current period minus the value of that variable in the previous period and is suggested to test the stationarity of the variables (Dickey and Fuller, 1979). The test results are shown in Table 2, which indicates that the variables after the difference are all stable, and the variables after the first-order difference passed the Augmented Dickey-Fuller (ADF) test and the Phillips-Perron (PP) test. Therefore, this paper used the first-order difference variables to analyze the subsequent VAR model.

**Table 2:** Stationary test.

First difference	Test type (C, T, L)	ADF test	PP test	Conclusion
$D.m\_pt_{t}$	(C,0,5)	−13.990^***^	−49.123^***^	Stationary
$D.m\_uct_{t}$	(C,0,5)	−14.338^***^	−46.159^***^	Stationary
$D.m\_\mathrm{fc}t_{t}$	(C,0,5)	−11.283^***^	−49.429^***^	Stationary
$D.v\_pt_{t}$	(C,0,5)	−13.532^***^	−51.531^***^	Stationary
$D.v\_uct_{t}$	(C,0,5)	−14.501^***^	−47.329^***^	Stationary
$D.v\_\mathrm{fc}t_{t}$	(C,0,5)	−7.282^***^	−34.665^***^	Stationary

(C, T, L) represents the constant term (a term that contains only a number), trend term (a term that reflects the influence of time trend), and lag term (a term that reflects the influence of the previous period to the current period) in the ADF test, and 0 means no item. The fifth-order lag term is the default lag order of the PP test, and the fifth-order lag term is also used in the ADF test. In addition, we also conducted the ADF test and PP test of trend items with 1 to 10 lags, and the conclusions obtained are consistent with this Table 2. The null hypothesis of the ADF test and PP test is that there is a unit root, *** representing a 1% significance level.

### Lag order selection

The general form of the VAR model was as follows (the coefficient before the variable is omitted, ε is the disturbance term):


(4) D.m_pttD.m_ucttD.m_fcttD.v_pttD.v_ucttD.v_fctt=D.m_ptt−1D.m_uctt−1D.m_fctt−1D.v_ptt−1D.v_uctt−1D.v_fctt−1+D.m_ptt−2D.m_uctt−2D.m_fctt−2D.v_ptt−2D.v_uctt−2D.v_fctt−2+…+D.m_ptt−qD.m_uctt−qD.m_fctt−qD.v_ptt−qD.v_uctt−qD.v_fctt−q+ε1tε2tε3tε4tε5tε6t

In Equation (4), q represented the order of lag. As previous literature suggested (Wang et al., 2020), it is common to choose q according to the AIC criterion. In this study, the corresponding AIC sizes of different lag orders q are shown in Table 3. It can be seen that the VAR model should be lagging fourth-order (q = 4) as the AIC value was the smallest in this order, which indicates that all variables are substituted into the analysis using values with a four-period lag.

**Table 3:** VAR lag order selection (AIC value).

Lagged order	1st order	2th order	3th order	4th order	5th order
AIC value	−11.537	−12.237	−12.388	−12.494	−12.406

### Model back testing

Although the form of the VAR model has been determined according to the AIC criteria, whether the model was reasonable still needs to be further tested. There are two crucial back testings for the VAR models: one is the autocorrelation test of the disturbance item ε as the disturbance term of the VAR model cannot have an autocorrelation problem (Johansen, 1995). Otherwise, it is necessary to adjust the model (e.g., adjust the lag order). The LM (Lagrange Multiplier) test can be used for verification. The test’s null hypothesis is that the disturbance term does not have an autocorrelation problem. The second is the stability test of the entire VAR model (Lütkepohl, 2005). The stability of the VAR model refers to the influence of a variable change on the variables in the VAR model that will gradually disappear over time. However, if this effect does not disappear, the VAR model needs to be re-adjusted. This stability can be judged according to whether the roots of the characteristic equation corresponding all fall within the unit circle or not. The VAR model is stable if all the roots fall within the unit circle. The autocorrelation test results of the disturbance term are shown in Table 4. From the lag one order to the lag four order, the value of p is greater than 0.5, which showed no evidence for autocorrelation in the model with the fourth-order lag.

**Table 4:** LM test of autocorrelation of VAR disturbance.

Disturbance term lag order	Chi-square value	Degree of freedom	*p*-value
1	41.243	36	0.252
2	45.328	36	0.137
3	41.442	36	0.245
4	37.312	36	0.409

**Figure 1:**
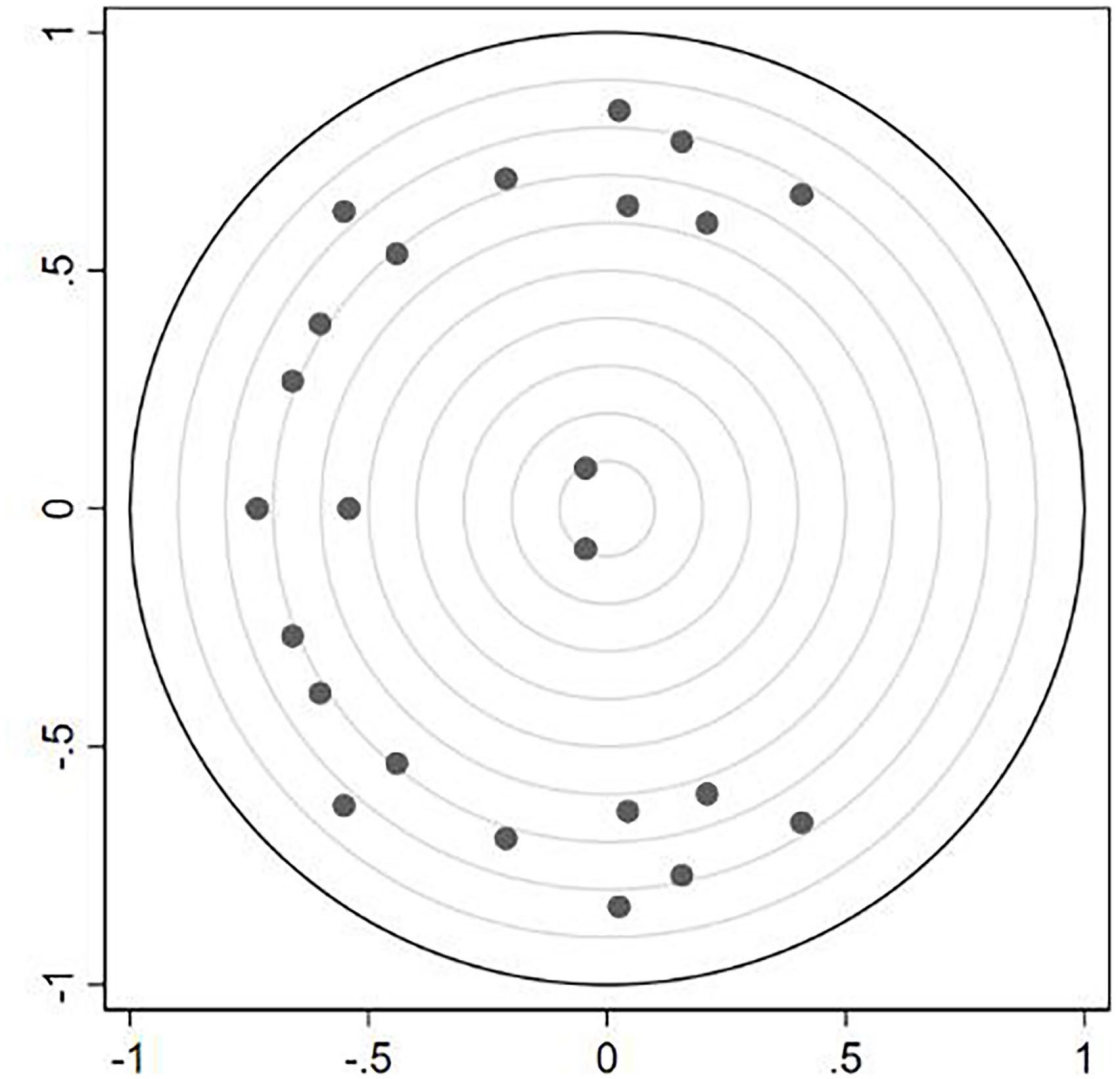
The stability condition of VAR. X-axis represents real, Y-axis represents imaginary.

The stability test of the VAR model is shown in Figure 1. As required, all the eigenvalues lie inside the unit circle, which implies that the estimated model is dynamically stable after the fourth-order lag.

From the results of the autocorrelation test of disturbance term and VAR stability condition test, it can be considered that the VAR model used in this study was reasonable and reliable.

## Results

### Impulse response function results

The impulse response function (IRF) describes how the response variable will change in the subsequent periods after the current period’s impulse variable increases by one unit of standard deviation. IRF has been widely used in many consumer behavior studies based on the VAR model (e.g., Srinivasan et al., 2016) as it can eliminate the effect of other variables and check the correlation between the two concerned variables. By analyzing the IRF, we can get a comprehensive insight into each impulse variable’s influencing direction and magnitude on the response variable in the VAR model. In the next section, this paper will analyze the cross-influence of the emotional valence and volatility of three sources (members’ posts, other members’ feedback, and focal firms’ feedback) through IRF for the subsequent ten periods (days). The emotional valence and volatility of members’ posts would be the response variable, and all the six variables would be the impulse variables in the first subsection. Similarly, the emotional valence and volatility of other members’ feedback and focal firms’ feedback would be the response variables in the second and third subsections. For simplicity, the abbreviations will be used in the following paragraphs, and their corresponding meanings can be seen in Table 1

**Figure 2:**
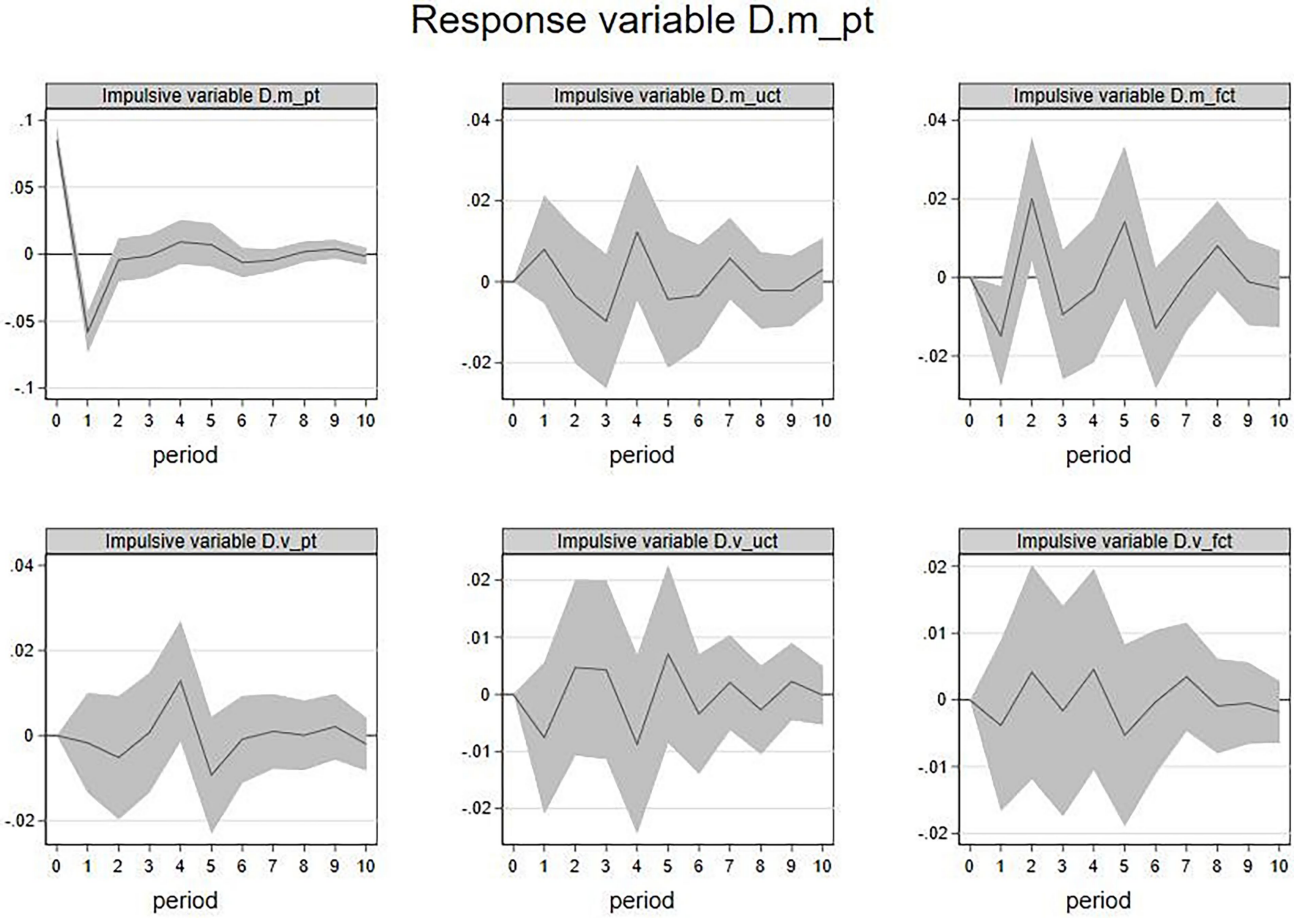
IRF: D.m_pt (response variable). Shadow represents 95% confidence interval. 2. X-axis represents period(s), and Y-axis represents the variation of the response variable. 3. The impulsive variable can be seen in the gray part at the top of each image. The same is below.

1. IRF of D.m_pt and D.v_pt . First, with members’ emotional valence ( D.m_pt ) as the response variable, this paper conducted the IRF analysis. The results are shown in Figure 2. We take the image in the upper left corner as an example to show how to interpret such an image. This image represented the influence of the current emotional valence of members’ posts on the future emotional valence of members’ posts in the following ten periods. The image showed a clear decline trend from the 0th period to the 1st period, indicating that the current emotional valence would negatively influence the emotional valence in the next period. It also showed an evident incline trend from the 1st period to the 2nd period, showing that the current emotional valence would slightly and negatively influence the emotional valence in the near future. This line tended to approach zero in the 3rd period, indicating that this effect will begin to fade at this time point. Similarly, the current period D.m_fct had a significant impact on the future D.m_pt . An increase in D.m_fct in the current period is likely to show a decline in D.m_pt in the next period but an increase in the future second period. The above results indicated that the increase in members’ emotional valence has a lagged negative impact on itself. The increase in focal firm feedback’s emotional valence in the current period is likely to show a rise and a fall alternately for members’ emotional valence in subsequent periods. However, these effects were short-lived.

**Figure 3:**
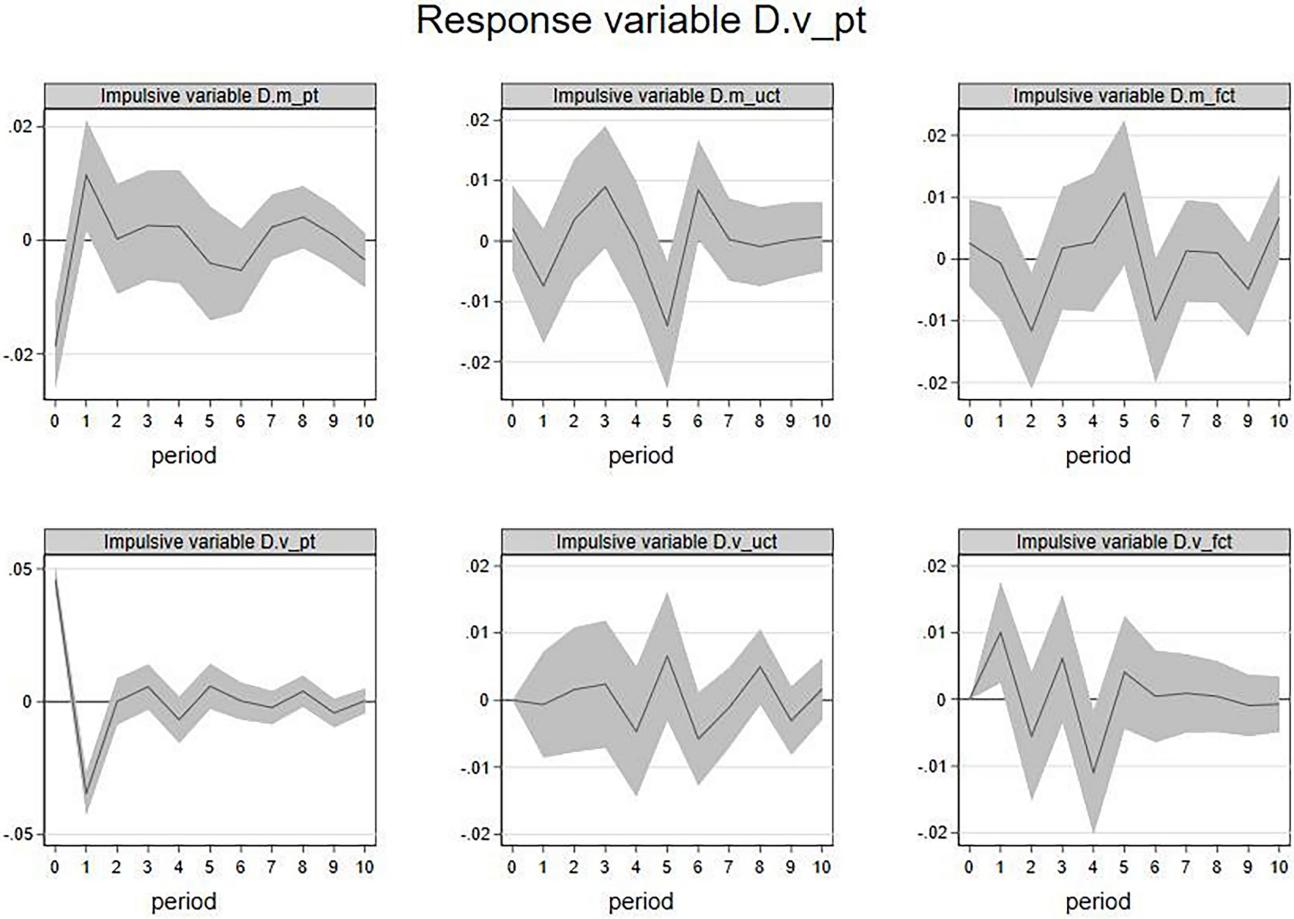
IRF: D.v_pt (response variable).

In the same vein, this paper analyzed the IRF when the members’ emotional volatility $\left(D.v\_\mathrm{pt}\right)$ was the response variable, and the results were shown in Figure 3. D.m_pt , D.m_uct , D.m_fct , and D.v_pt in current period have a significant impact on D.v_pt in the future. Among them, the increase of D.m_pt in the current period is associated with D.v_pt rise in the coming first period, the increase in D.m_uct in the current period is associated with D.v_pt fall in the fifth period in future, the increase in D.m_fct , in the current period is associated with D.v_pt fall in the second period, and the increase in D.v_pt in the current period will lead D.v_pt to fall in the first period. The increase in D.m_fct in the current period is likely to show a rise for D.v_pt in the first period but a decline in the fourth period in the future.

Above all, the members’ emotional volatility will be positively affected by the lag of members’ emotional valence, but it will also be negatively affected by the emotional valence of other members’ feedback and the firm’s feedback. In addition, it will also be affected by members’ emotional volatility. While that an increase in the firm feedbacks’ emotional volatility will first increase and then decrease members’ emotional volatility. Besides, the results showed that the duration of these effects is transient.

**Figure 4:**
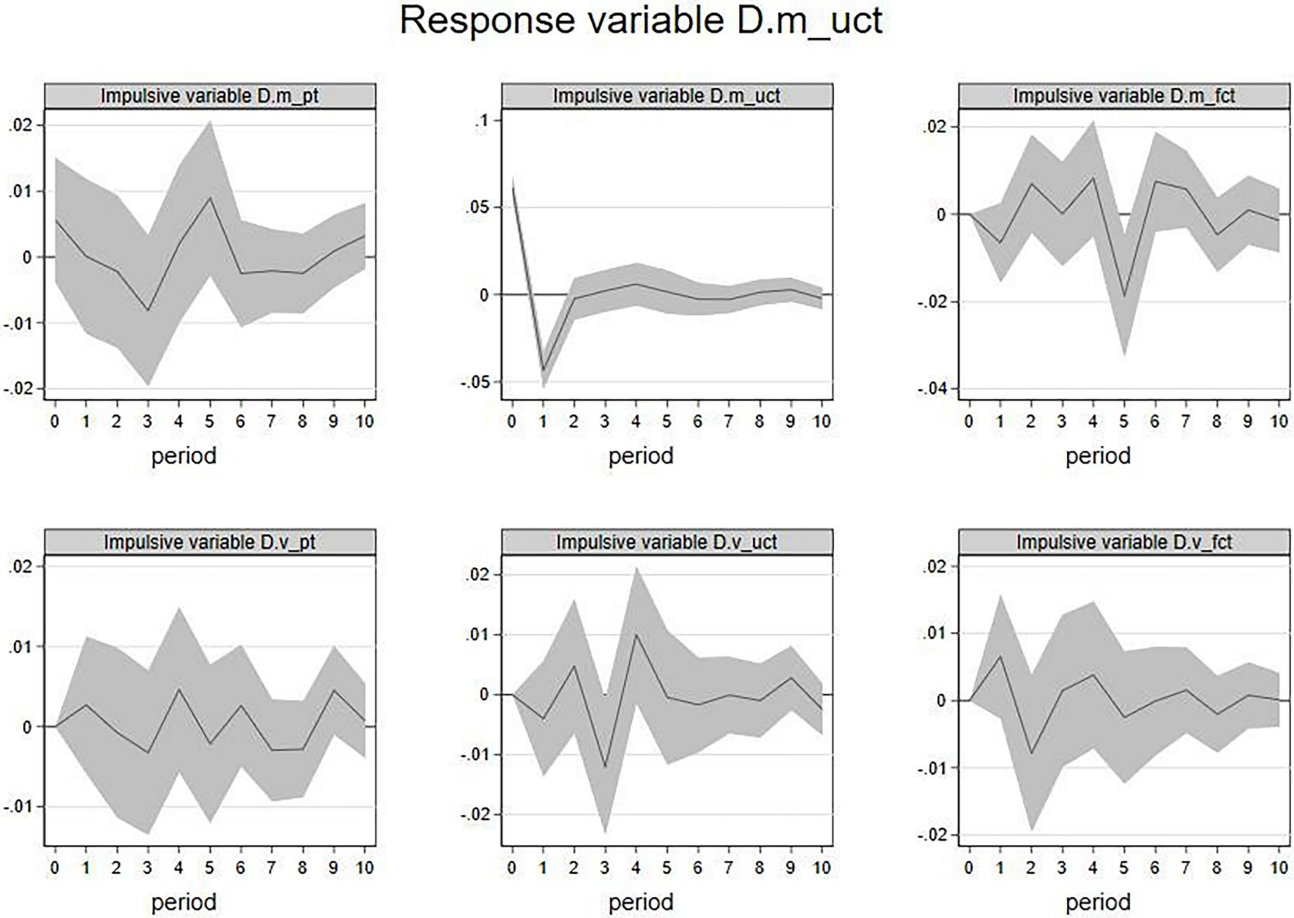
IRF: D.m_uct (response variable).

2. IRF of D.m_uct and D.v_uct . This paper analyzed the IRF when the emotional valence of other members’ feedback $\left(D.m_{uct}\right)$ as the response variable (Figure 4). The current D.m_uct , D.m_fct , and D.v_uct will influence future D.m_uct . An increase of D.m_uct in the current period is associated with D.m_uct decline in the first period, and the increase in D.m_fct in the current period is associated with D.m_uct decline in the fifth period, and the increase in current D.v_uct is associated with D.m_uct decline in the third period. The above results showed that the emotional valence of feedback from other members would be negatively affected by the lag of its own and be negatively affected by the emotional valence of the firm’s feedback and the emotional volatility of other members’ feedback. Nevertheless, no matter which kind of interaction, the duration is short.

**Figure 5:**
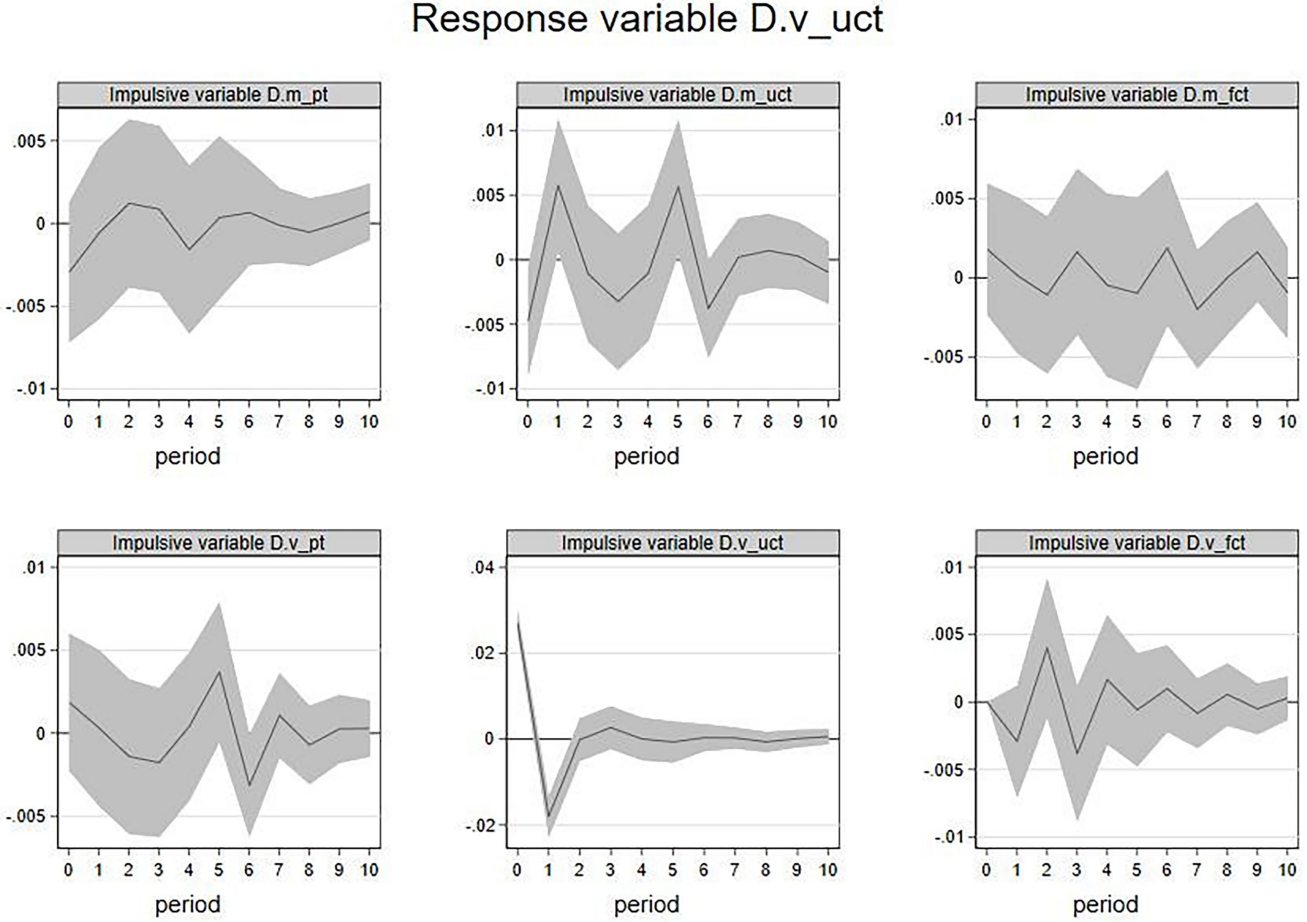
IRF: D.v_uct (response variable).

In addition, this paper analyzed the IRF when the emotional volatility of other members’ feedback $\left(D.v\_uct\right)$ as the response variable (Figure 5). current period D.m_uct , D.m_fct , and D.v_uct had impacts on future D.v_uct . An increase of D.m_uct in the current period is associated with D.v_uct fall in the 3rd period, and the increase in D.m_fct and D.m_uct in the current period is associated with D.v_uct fall in the first period. The above results showed that the emotional volatility of other members’ feedback would be negatively affected by the emotional valence of other members’ feedback, the emotional valence of the firm’s feedback, and the emotional volatility of other members’ feedback. Likewise, the duration of these effects was short-lived.

**Figure 6:**
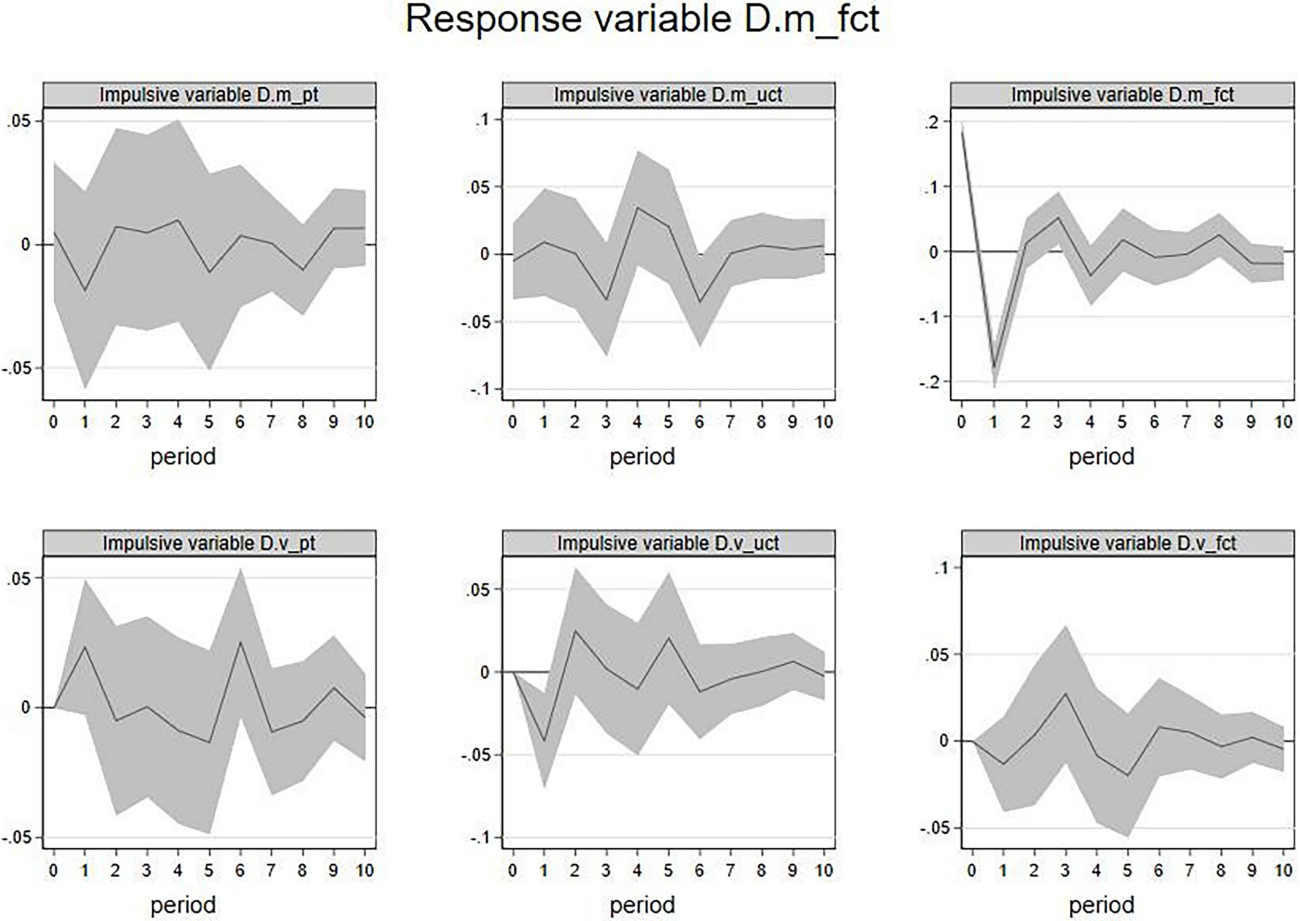
IRF: D.m_fct (response variable).

3. IRF of D.m_fct and D.v_fct . Figure 6 showed the results of IRF when the D.m_fct (emotional valence of firm’s feedback) was the response variable. The current D.m_uct , D.m_fct , and D.v_uct had significant impacts on the future D.m_fct . Among them, an increase in D.m_uct in the current period is likely to show a decline for D.m_fct in the next 6th period, an increase in D.m_fct in the current period is likely to show a decline for D.m_fct in the coming first period and a rise in the second period. An increase in D.v_uct in the current period is likely to show a decline for D.m_fct over the coming first period too. The above results indicated that the emotional valence of the firm’s feedback would be negatively affected by the emotional valence and emotional volatility of feedback from other members and negatively affected by the emotional valence of the firm’s feedback itself. Similarly, the duration of these effects is short.

**Figure 7:**
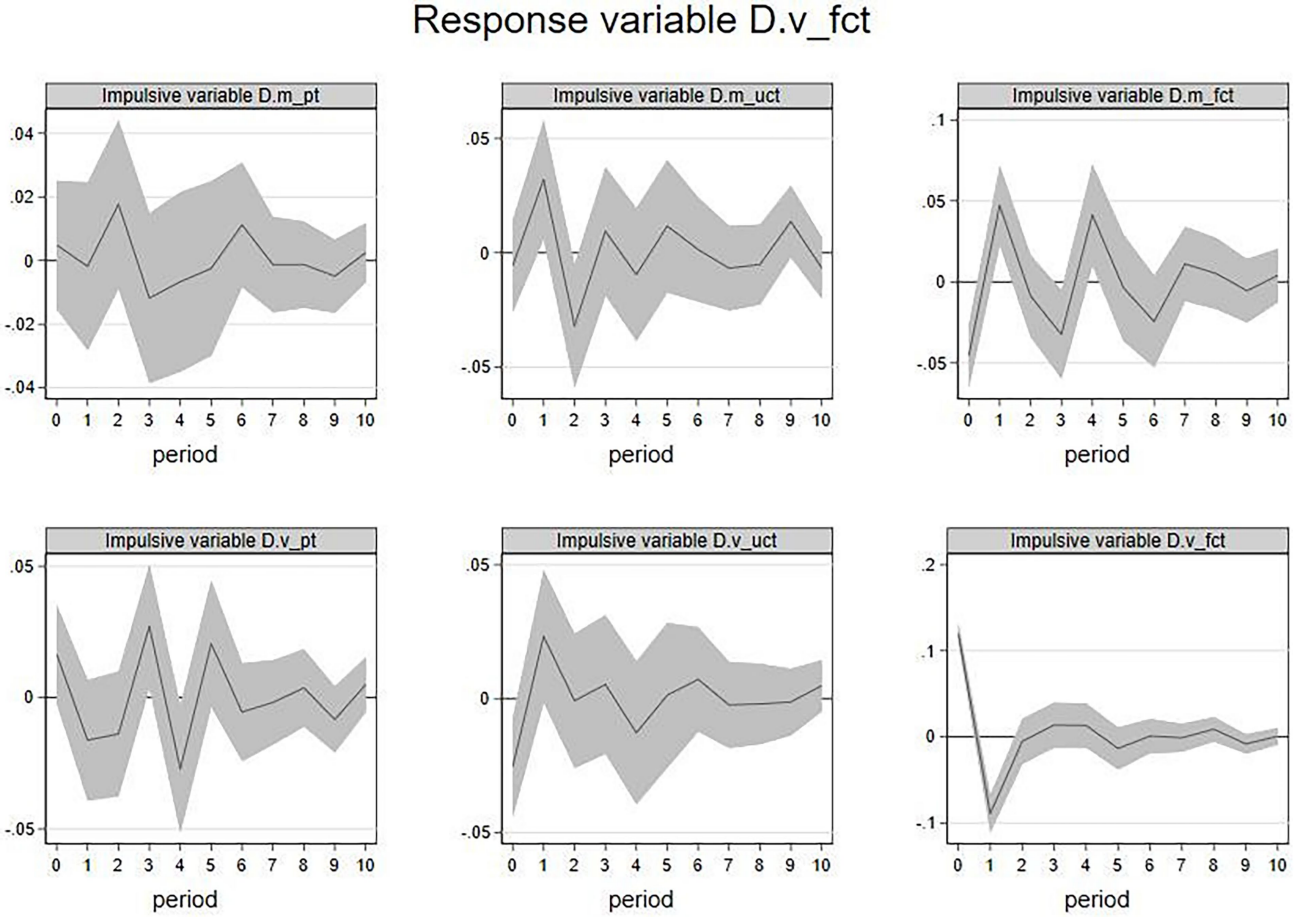
IRF: D.v_fct (response variable).

The IRF of the firm’s feedbacks emotion volatility $(D.v_{fct}$ ) as the response variable was shown in Figure 7. The current D.m_uct , D.m_fct , D.v_pt , and D.v_fct had impacts on the future D.v_fct . Among them, the increase in D.m_uct of the current period is likely to show a rise for D.v_fct in the first period but a decline in the second period. An increase in D.m_fct in the current period is likely to show a rise for D.v_fct in the 1st and 4th periods but a decline in 3rd period. The increase in D.v_pt in the current period is associated with D.v_fct rise in the 3rd period but fall in the 4th period. The increase in D.v_fct of the current period is likely to show a decline for D.v_fct in the coming first period.

The above results showed that the emotional valence of feedback from other members, the emotional valence of the firm’s feedback, and the emotional volatility of members had an alternating effect on the emotional volatility of the firm’s feedback. In contrast, the emotional volatility of the firm’s feedback had a lagging and negative impact on itself. Likewise, the duration of these effects was temporary.

### Variance decomposition results

The above analysis depicted the significance and direction of the mutual influence between different sources of emotion in both valence (mean) and volatility (dispersion). This section will move on to the VAR model’s FEVD to further analyze the mutual contribution rate between different sources of emotion. FEVD can quantitatively calculate all impulse variables’ contribution ratio that increases by one standard deviation in the current period to the mean square error (MSE) of the response variable in each future period.

For brevity’s sake, the FEVD results of each response variable in the 1st, 4th, 7th, and 10th period in the future was presented as examples. Since the results of these four periods are only numerically different, the order and importance of contributions remained unchanged. The contribution rate of each impulse variable on the response variable will be accordingly changed as the influence would change over time gradually. Thus, this paper will analyze each response variable’s primary and secondary contributors in the 10th period of FEVD in the following section.

1. FEVD of D.m_pt and D.v_pt . In line with the first subsection in the last part, the variance decomposition results when the emotional valence of members’ posts $(D.m_{\mathrm{pt}}$ ) and the emotional volatility of members’ posts $\left(D.v\_\mathrm{pt}\right)$ as the response variables were presented in Table 5. The two variables were mainly influenced by themselves, even if the influence is gradually fading over time. A standard deviation increase in the current period D.m_pt will contribute 83.3% to D.m_pt at the 10th period, while the increase of a standard deviation in the current period. D.v_pt will still contribute 65.4% to D.v_pt at 10th period. Except that the main contributors are all themselves, D.v_pt ’s secondary contributor was D.m_pt, and D.m_pt ’s secondary contributor was D.m_fct . In contrast, the increase of a standard deviation in the current period D.m_fct will contribute 8.8% of D.m_pt ’s variation in the tenth period, and the increase of a standard deviation in the current period D.m_pt will contribute 10.6% of D.v_pt ’s variation at tenth period D.v_pt . According to the above analysis results, the main contributor to variations in the emotional valence or volatility of posts was the variable itself, but the secondary contributor was firm feedbacks’ emotional valence.
FEVD of D.m_uct and D.v_uct . Similarly, according to Table 6, the main contributor to emotion valence or volatility of other members’ feedback was itself, but the secondary contributor of emotion valence of other members’ feedback was the emotional valence of firm feedback, and the secondary contributor of emotion volatility of other members’ feedbacks was emotional valence of other members’ feedback.
FEVD of D.m_fct and D.v_fct . As shown in Table 7, the main contributor to firm feedback’s emotional valence or volatility was itself. However, the secondary contributor to the firm feedback’s emotional valence was other members’ emotional valence, while the secondary contributor to firm feedback’s emotional volatility was firm feedback’s emotional valence.

**Table 5:** The source of contribution ( D.m_pt or D.v_pt as response variable).

Response variable	Period impulse variables	1st	4th	7th	10th
D.m_pt	D.m_pt	100.0	91.1	84.2	83.3
	D.m_uct	0.0	1.5	2.8	3.0
	D.m_fct	0.0	6.1	8.4	8.8
	D.v_pt	0.0	0.3	2.2	2.2
	D.v_uct	0.0	0.8	1.8	1.9
	D.v_fct	0.0	0.3	0.7	0.7
D.v_pt	D.m_pt	14.1	11.3	10.4	10.6
	D.m_uct	0.2	3.5	8.1	7.9
	D.m_fct	0.3	3.3	7.0	7.4
	D.v_pt	85.5	77.7	66.4	65.6
	D.v_uct	0.0	0.2	2.1	2.7
	D.v_fct	0.0	3.9	5.9	5.8

The units in the table are percentages. For example, the 5.8 in the bottom right corner of the table indicates that the contribution rate of focal firms’ emotional volatility to members’ emotional volatility in the tenth period is 5.8%; 2. The meaning of the abbreviations in this table can be seen in Table 1. The same is below.

**Table 6:** The source of contribution ( D.m_uct or D.v_uct as response variable).

Response variable	Period impulse variables	1st	4th	7th	10th
D.m_uct	D.m_pt	0.8	1.7	2.8	2.9
	D.m_uct	99.2	91.8	82.4	81.0
	D.m_fct	0.0	1.5	8.1	8.8
	D.v_pt	0.0	0.3	0.7	1.2
	D.v_uct	0.0	3.0	4.2	4.2
	D.v_fct	0.0	1.7	1.8	1.9
D.v_uct	D.m_pt	1.1	0.9	1.1	1.1
	D.m_uct	2.9	5.6	8.9	8.9
	D.m_fct	0.4	0.6	0.9	1.4
	D.v_pt	0.5	0.7	2.5	2.6
	D.v_uct	95.0	88.8	83.1	82.4
	D.v_fct	0.0	3.3	3.4	3.5

**Table 7:** The source of contribution ( D.m_fct or D.v_fct as response variable).

Response variable	Period impulse variable	1st	4th	7th	10th
D.m_fct	D.m_pt	0.1	0.6	0.8	1.0
	D.m_uct	0.1	3.5	8.1	7.9
	D.m_fct	99.8	92.5	86.7	86.4
	D.v_pt	0.0	0.8	1.8	2.0
	D.v_uct	0.0	3.2	3.7	3.7
	D.v_fct	0.0	1.3	1.8	1.8
D.v_fct	D.m_pt	0.1	1.4	1.7	1.8
	D.m_uct	0.2	6.5	6.3	6.9
	D.m_fct	12.0	16.4	20.6	20.7
	D.v_pt	1.6	4.4	7.0	7.1
	D.v_uct	3.7	3.7	3.8	3.8
	D.v_fct	82.3	67.6	60.5	59.8

## Discussion

### Main findings

Although emotional contagion is ubiquitous in the virtual environment (e.g., Lin and Utz, 2015), few scholars have conducted in-depth studies on the emotional contagion process between members, other members, and community administrators in the OBCs setting. Therefore, this study was formally analyzed in Xiaomi community that focuses on technology products (e.g., mobile phones). The members of this kind of community are mainly consumers of that type of product, so its conclusions revolve around the consumer goods community as well. To further understand the emotional cross-influence dynamically, based on emotional contagion theory and the VAR model, this study divided the mutual influence of sentiment from different sources into two dimensions: valence and volatility. The VAR model is designated as the “reduced form” of a structural model in its most general form and has been used to detect causality in many fields (Lütkepohl, 2005). The nature of estimating the dynamic cross-relationships of all the endogenous variables suits the emotional contagion in this study. This study further analyzed the interaction between the emotional valence and volatility of members’ posts, other members’ feedback, and the firm’s feedback in OBCs. Specifically, this study examined the directions and magnitude of different effects among the three sources of emotions and drew the following findings.

Regarding the directions of the mutual influence between different sources of emotions, this study showed: (1) Different sources (e.g., members’ posts) of emotion have different directions of mutual influence on two emotional dimensions (valence and volatility). For example, an increase in the emotional valence of firm feedback in the current period is likely to show a rise in the emotional valence of members’ posts first and then a subsequent decline, but it will also reduce the emotional reaction of other members’ feedback. (2) Each source’s emotion significantly influences itself in terms of valence or volatility. For example, an increase in the emotional valence of current members’ posts is likely to show a decline in the emotional valence of members’ posts in the future. An increase in the emotional volatility of current members’ posts will reduce the emotional volatility of future members’ posts. (3) Despite differentiated cross-influences, the duration of the mutual influence of emotion from different sources was very short, and most did not exceed three periods (days).

Regarding the magnitude of mutual contribution between different sources of emotion, this study indicated: (1) In a relatively long period, the most critical contributor to variations of emotion was itself. (2) The secondary contributor to the emotional valence of members’ posts and other members’ feedback was the emotional valence of firms’ feedback, while the secondary contributor to the emotional valence of firms’ feedback was the emotional valence of other members’ feedback, which indicated that the focal firm’s actions could explain the variations in emotional valence of members and repliers. (3) The secondary contributor to different sources’ emotional volatility was their emotional valence. For example, the valence was the secondary contributor to the emotional volatility of members’ posts, indicating that the valence can be an essential interpretation for variations in emotional volatility.

In sum, this study showed that different sources of emotion had different directions of mutual influence on both emotional valence and volatility, and each source’s emotion had a significant negative influence on itself in terms of valence or volatility. This study also found that the most critical contributor to variations of emotion was itself in a relatively long period. The emotional valence of firms’ feedback exerted considerable influence on the emotional valence of members’ posts and other members’ feedback.

### Theoretical contributions

The main theoretical contributions of this study are as follows: First, the importance of emotion and its formation were highlighted in OBCs research (e.g., Flavián et al., 2011; Belanche et al., 2019; Anaya et al., 2020). However, few scholars have empirically studied whether there is an interaction between emotions *per se* in OBCs. Considering the bilateral interaction between community members and official staff (Gruner et al., 2014), this study further distinguished three emotional sources in OBCs: members who put the posts (members’ posts), other members who reply to the posts (other members’ feedback), and firm’s staff who reply the posts (firm’s feedback), which was barely considerate in previous research, echoing to emotion spillover effect proposed by Yegiyan (2015) and extending this effect in the online environment. This study enriches the emotion research in the OBCs context to a certain extent through a second-hand data study and contributes to community members’ emotional management in OBCs.

Second, this study distinguished the emotional valence and volatility, which helps to understand the emotional cross-influences in OBCs comprehensively and deeply and expands the applied scope of the emotional contagion theory by providing new empirical shreds of evidence in this stream of research. Existing studies on emotional contagion mainly focus on the mutual influence of emotional valence (e.g., Rasmussen and Berntsen, 2009; Adam et al., 2016), while ignoring that emotional fluctuation also exists within interpersonal interactions in the virtual environment (Cabrera et al., 2018). This study shows that emotional volatility plays a vital role in the process of emotional contagion, which is an extension of the emotional contagion theory.

### Practical implications

This study also provides guidance to firms that aim to build and develop their OBCs. This research found that the most significant influencer of each source’s emotion is itself. Thus, special attention to the emotional atmosphere in OBCs is suggested for firms. Firms should encourage the members to use positive words in posting or replying because of the lasting effect of the current emotional valence of vitality. Firms can also use “common words” in OBCs to lead the members to use similar words. For example, when responding to a member’s technical query about a product, the firm should use less specialized technical terms to respond, and instead convert those terms into layman’s language that the average person can understand. Consequently, the emotional atmosphere will be harmonious, leading the members to form trust and loyalty toward the firm (Anaya et al., 2020).

Furthermore, this study also helps firms manage or influence the members’ emotional status in OBCs. Firms can cultivate positive emotions among members by dynamically adjusting their management behaviors toward the community. Thus, firms can use words that contain positive emotions or make object replies to reduce the emotional fluctuation when providing feedback to members’ posts, significantly promoting a positive and stable emotional environment in OBCs. For example, when replying to a post, firms should use polite words such as “please,” “glad” and “happy.” This includes adding “I’m happy to answer your question” to a reply to your post. In addition, firms can hire some proactive staff to manage the OBCs, and this can speed up the emotional interactions between members and firms. This study also suggested that the firms need to “monitor” the sentimental changes in OBCs (Akdim, 2021) and engage in the communities frequently. Firms can use opinion analysis tools to probe members’ sentiments and actively intervene at the right time to guide the healthy development of the community.

### Limitations and future research

This study has some limitations, and many potential works could be done in future research. First, this study used the MIUI forum as the empirical context to study the mutual influence of emotion from different sources. This forum was launched and managed by Xiaomi, and technology products are the main business of this company. Although emotional contagion occurs in various kinds of online communities, there may be differences in the process of emotional contagion. For example, changes in celebrity sentiment may play a dominant role in members’ sentiment in celebrity communities. Future research can be based on results in this paper to further discuss the heterogeneity of other communities. Second, this study was based on the overall perspective by analyzing the time-series data to detect the cross-influences. Although a VAR model was proved to be an effective tool in analyzing such problems, future studies can still consider obtaining more refined data (e.g., data with personal information) to conduct field experiments to obtain more robust results when conditions permit. Third, this study avoided significant events that may occur in OBC when selecting data, but what effect these significant events have on the mutual influence of emotion from different sources is a practically important topic. Future research can extend current work by applying critical incident techniques or difference-in-difference (DID) estimation.

## Data availability statement

The original contributions presented in the study are included in the article/Supplementary material, further inquiries can be directed to the corresponding author.

## Author contributions

DL contributed to conceptualization, investigation, writing, and visualization, review, and editing. SZ and QL contributed to the substantial revision. DL successfully applied for the sponsorship of our research. SZ contributed to conceptualization, methodology, software, formal analysis, and writing. All authors contributed to the article and approved the submitted version.

## Funding

This work was supported by the General Project of Philosophy and Social Science Research in Jiangsu Universities under grant 2022SJYB0103 and the Talent Introduction Project of Nanjing University of Posts and Telecommunications under grant NYY222011.

## Conflict of interest

The authors declare that the research was conducted in the absence of any commercial or financial relationships that could be construed as a potential conflict of interest.

## Publisher’s note

All claims expressed in this article are solely those of the authors and do not necessarily represent those of their affiliated organizations, or those of the publisher, the editors and the reviewers. Any product that may be evaluated in this article, or claim that may be made by its manufacturer, is not guaranteed or endorsed by the publisher.
